# Gonorrhœa*Read before the Buffalo Medical Club, October 1st, 1879.

**Published:** 1879-11

**Authors:** Geo. W. Stoner

**Affiliations:** Asst. Surgeon U. S. M. H. S.


THE BUFFALO

### MEDICAL AND SURGICAL JOURNAL.

VOL. XIX.—NOVEMBER, 1879.—No. 4.

### Original Communications.

### GONORRHŒA.*

BY GEO. W. STONER, M. D., ASST. SURGEON U. S. M. H. S.

Gonorrhœa, or the disease popularly known as such, is not what the term in its etymological sense implies, it being derived from two Greek words, signifying a flow of semen, and as the urethral discharge usually distinguished by the above title contains no semen, it is quite clear that the term as used is a misnomer, just as much so as gastrotomy is for the Cæsarean section.

It would be preferable, therefore, to use the term specific urethritis, as distinguished from simple urethritis, which is different in character, and caused, as a rule, by means other than direct contagion from the vaginal discharge of a woman suffering from the virulent disease.

Specific urethritis is always produced by direct contagion; simple urethritis may be, and frequently is, but not always, as will be shown further on. But in order to avoid confusion, and inasmuch as long usage has given the term gonorrhœa a more or less definite signification, it may be well to continue the use of it, as applied to the specific urethral inflammation, and restrict

* Read before the Buffalo Medical Club, October 1st, 1879.

the word urethritis to the simple inflammation. Clinically, however, it is very difficult, if not impossible, to distinguish one from the other, and, as the treatment is essentially the same in either case, it is of little importance, practically, so far as the management of a given case is concerned. But there are other and better reasons why the distinction should be recognized. Gonorrhœa is urethritis, but urethritis is not necessarily gonorrhœa. And for this reason it is very important that the two diseases should be known by names very dissimilar, notwithstanding the fact that in the majority of cases the difference is not apparent.

Granting that simple urethritis, simulating gonorrhœa, is comparatively rare, we must not lose sight of the fact that such cases do occur; and to accuse a man or woman of having gonorrhœa, is, of course, asserting his or her infidelity, and may be the starting point of heaping accusation after accusation upon the innocent, and the cause of much unhappiness. Gonorrhœa ought not to be a disease of the married, nor indeed of the unmarried; unfortunately, however, it presents itself in all classes, married and unmarried, rich and poor, high and low, the preponderance, however, being largely on the side of the unmarried, and I have seen a case in a boy not more than twelve years old. Men who frequent houses of prostitution, or cohabit with street-walkers, are indeed fortunate if they contract nothing more serious.

The earliest history of urethral disease, of which we have any knowledge, is that contained in the fifteenth chapter of Leviticus, which probably embraces both varieties. Those who contend that the disease of that age was the simple urethritis have no good grounds for such *conjecture.* Were the habits, surroundings, and condition of the people of that age better, physically and morally, than they are of people of to-day? Is it not more reasonable to believe that the "*running issues*" of that time were, to a great extent, made up of what we at present know under the name of gonorrhœa? At any rate we are furnished with good practical advice in the chapter referred to.

Gonorrhœa is a local disease, a specific urethritis, notoriously contagious and usually acquired by coming in direct contact with a person having it. Any or all exposed mucous membranes may be the seat of the disease; all that is necessary to produce it, is simple contact in the sexual act or otherwise. Clinical experience goes to show however that gonorrhœal inflammation, in parts other than the sexual organs, is not very common. Gonorrhœal conjunctivitis being perhaps the most frequent, and this, according to Bumstead, occurs only once in about six hundred cases. It is said to be more frequent in the male than in the female, and oftener in the right eye than in the left, as most patients (according to recent writers on the subject) handle the penis and rub the eye with the right hand. Occasionally, however, both eyes are affected, as I have recently had a case under treatment, but in this patient the disease was acquired by using a towel, on board ship, that had been used by another suffering from gonorrhœa, the patient himself having no urethral discharge whatever.

But, to continue the subject of gonorrhœa and consider its history clinically: The disease as has already been stated is always caused by contagion, and usually makes its appearance about five days after exposure, but may occur as early as twenty-four hours or as late as two weeks. It is stated by some authors, that the shorter the period of incubation the milder will be the attack, but to this there are too many exceptions to state it as a rule. It generally commences by an itching or tickling about the orifice of the urethra and a sensation of heat in voiding urine; the lips of the meatus are found more or less swollen, which gradually and sometimes rapidly increases; between them is noticed a thin, sticky and bluish looking discharge, which, as the inflammation increases, becomes more copious, and of a muco-purulent or purulent character, and then changes to the thick yellow or yellowish-green which is sometimes mixed or streaked with blood. The swelling of the lips and mucous membrane of the urethra increase, and, if the inflammation be very acute at about the sixth or seventh day, the whole glans and penis will be found swollen, and the urethra so red and sensitive that the slightest erection causes pain, and the act of urination is attended with intense scalding, while the stream is small or dribbling, the passage being obstructed by the swelling, and the mucous membrane eroded. Pain in the back and perineum is now also felt. Retention of urine may take place by spasmodic contraction or by extension of the disease to the prostate; but as the disease begins at the meatus and travels backwards, and, according to Desmoreaux, only reaches the middle of the urethra about the eighth day, retention will not follow, if proper treatment is commenced in time; at any rate, retention other than spasmodic is very rare in gonorrhœa, except in those cases where stricture previously existed. Involuntary seminal emissions are frequently caused by the local irritation, and are attended with sharp pain. As the disease advances, the symptoms become more severe, the perineal pain increases, and by extension or infiltration from the mucous membrane of the urethra into the meshes of the corpus spongiosum, the areolar structure becomes obliterated by the effusion of lymph, and the erectile tissue has not sufficient room to expand; as a consequence, very painful erections or chordee come on, and according to the extent and location of the inflammatory action or deposit, there will be a bending of the penis downward, like a bow, during erection; the urethra being apparently too short for the corpora cavernosa. If on the other hand the corpora cavernosa become the seat of inflammation, and the corpus spongiosum be not effected, the bending will be in the other direction, as I have recently had a case under treatment,—but this is rare. These painful erections or chordee are of course most frequent during the night and toward morning. The groin and testicles or rather the epididymis are also frequently involved. Inflammation of the inguinal glands with, in neglected cases, suppuration also occurs, as well as phymosis and paraphymosis. In some cases there is a general febrile action of considerable height, but in most cases it is absent. After the lapse of ten days to three weeks, the acute symptoms may partially subside, and very much earlier if judicious treatment be resorted to in time; the discharge will become thinner, the heat and redness less marked, and the pain on urination gradually ceases, and the chordee becomes less frequent. Finally the discharge becomes very thin and watery and diminishes in quantity, till a single drop is noticed in the morning, and this, too, soon ceases. In unfavorable cases, however, the disease, as it goes on toward recovery, stops at the watery or muco-purulent stage, and keeps up a continual discharge, and to this, the name of chronic gonorrhœa, or gleet, has been applied. In many cases, gleet is caused and kept up by a contraction or stricture of the urethra, which is best treated by overcoming the stricture. But there are also many cases of chronic urethral discharge following gonorrhœa, in which very little, if any, real stricture can be detected. And according to the dissections of Sir Astley Cooper and Ricord, the seat of the disease is in the fossa navicularis and the lacunae. The disease, according to some authors, may extend from the urethra to the bladder, decreasing at the point of origin as it extends inwards, but this is very rare.

Gleet is kept up, in my judgment, in many cases by the diseased condition (the low grade of inflammation) that remains in the lacunae after the discharge from the urethra proper has ceased, where the thin watery or slightly purulent discharge continues from the mouths of these little cavities. If on the other hand the mouths of the lacunae become closed by the inflammation, and the discharge is occluded, a cyst is produced, which as a rule enlarges, softens, and finally comes to the surface where it can be treated by enucleation; the same as cysts in any other location, they seldom, if ever, open into the urethra. Abscess of the penis, caused by suppurative inflammation, occasionally occurs, and may involve the whole of the penis, and if it begins at the bulb or glands of cowper, as is rarely the case, it may involve the whole perineum and scrotum.

If ulceration into the urethra takes place, the case becomes more serious; retention and infiltration of urine and the burrowing of the matter, all tend to increase the gravity of the case, and require close attention. The operation of external urethrotomy may become necessary. Inflammation of the lymphatic vessels is quite common in severe forms of gonorrhœa, and, if the inflammation extends outside of these vessels, reddened streaks, very sensitive to the touch, are seen on the sides and back of the penis, and the vessels feel like hard and knotty cords; the glands become hard and painful, and the prepuce œdematus.

In some cases the lymphitis is limited to the superficial lymphatics of the prepuce, where there is a general diffused redness and œdema, and as a rule, troublesome phymosis. If suppuration occurs, it is to be treated by early incision to prevent burrowing, which, of course, also applies to all other forms of suppuration in these parts.

Other important complications or sequelae of gonorrhœa are stricture of the urethra, gonorrhœal rheumatism, gonorrhœal ophthalmia, etc., but these, in themselves, are of much importance, and could not even be hinted at in a paper of this kind. Before entering, however, upon the practical part of the subject, (viz: the treatment of gonorrhœa or urethritis) and inasmuch as it is so important not to pronounce too hastily upon the character of a given case, it is desirable to have some understanding as to what constitutes the real, although not apparent, difference between gonorrhœa and simple urethritis.

The difficulty, and indeed the impossibility of distinguishing the true character of a urethral discharge, is fully recognized by all the best writers.

Van Buren and Keyes state emphatically that gonorrhœa cannot be separated from urethritis clinically, and that a urethritis presenting absolutely nothing to differentiate it from gonorrhœa may be acquired by a healthy young lover from his equally healthy mistress, by a young husband from his wife, and that it may be produced by applying chemicals, or by introducing any abnormal and irritating substance into the urethra. All agree that the menstrual flow, and especially leucorrhœal discharges coming in contact with the mucous membrane of the penis may be the cause of urethritis, provided the system at the time of contact be in a condition for its propagation, or the urethra be in a damaged condition (the result of previous inflammations) or the urine be very acid and irritating, or some individual idiosyncracy. Persons of a gouty or rheumatic constitution are liable to suffer from these causes. According to Simon, all inflammations, specific or otherwise, are communicable; that pus from a given inflammation is always, in its kind, to some extent, contagious. And Diday states that "the very fact of a woman having a discharge, no matter what its origin, she is liable to give a discharge to a man," which means of course to some men, for it is a notorious fact that one man may perform the sexual act with impunity with a woman having a discharge while a second immediately following him may become affected. This applies, however, only to the discharge of the simple character, and is explained by the fact of the second man having a large meatus, and being under stronger venereal excitement, and probably full of wine. The second man, however, according to Ricord, may soon become "acclimated," so that later on he may live with the same woman, having the same discharge, without contracting it.

Ricord's "receipt for getting a gonorrhœa," (or rather a urethritis) is based largely upon these conclusions, and consists in exciting the animal passions of both man and woman by wining and dining, together with dancing (round dancing) and performing the sexual act as often as possible during the night, and taking a prolonged warm bath, and a "precautionary" injection in the morning.

The treatment of gonorrhœa is frequently divided under two heads, the abortive and the methodic treatment, but this, it seems to me, is unnecessary, if not incorrect. The abortive treatment is to a certain extent methodic, and the methodic treatment is directed towards aborting the disease—using the term in its broadest sense, *i. e.*, cutting short or thwarting the disease from its natural course and bad tendency.

The abortive treatment, or the "burning out" of the disease, as it was originally called, with a strong solution of nitrate of silver (10 to 40 grs. to f℥i) has long since been condemned as not only a useless, but a pernicious practice. Nothing, it seems to me, could be worse, except the solid stick or the stronger acids, than to inject a 40 gr. solution of nitrate of silver into an inflamed urethra; and here the question may arise as to the reason why such treatment cannot, with the same degree of practicability or impunity, be resorted to as in the treatment of mucous membranes elsewhere, as for instance, in the eye, the most delicate and sensitive of all organs, which may be fully answered by simply reviewing the anatomy of the parts.

Astringent injections are very important, and in my opinion the most important, as remedies in the management of a given case of gonorrhœa; but if nitrate of silver is used, it must be with caution, and in a very weak solution at that. There are other and better remedies. In gonorrhœa, as in some other diseases, we occasionally meet with cases that the usual good remedies given alone fail to benefit. Quinine is the remedy for ague, and is sometimes called a specific for it, yet we occasionally meet with cases that it does not control, and where arsenic plays a better part. In most cases of ague, quinine has a better effect when combined with a small quantity of opium, and in many cases is much aided in its effect, or the system is better prepared for its action by the administration of a mercurial cathartic or podophyllin.

There are, indeed, few articles of the materia medica that have not at some time or another been used in the treatment of ague or malarial fever, and this is equally true of the disease known as gonorrhœa. Copaiba, cubebs, oil of sandal wood, and a host of other articles have from time to time been used, and are still the principal remedies recommended by comparatively recent authors, for internal administration; and granting that they are of considerable value in the treatment of many cases, especially if combined with an alkali, and properly administered, they are in my judgment, unnecessary in many, if not most cases. Nearly all the astringents of the materia medica have from time to time been used as injections for gonorrhœa, but so far as my experience goes, nothing, that is to say *no one thing*, is so useful as the sulphate of zinc; this alone in a solution of two to four grains to an ounce of water, or preferably rose water, or as I am in the habit of prescribing it: one to two grammes to two hundred and fifty cubic centimetres of water, will effect a cure in some cases; but if we combine the sulphate of zinc with tannic acid, in the proportion of two grammes of the former to one-half to two grammes of the latter in two hundred and fifty cubic centimetres of water, we obtain an astringent solution which in my experience has produced better results than any of the many other solutions and combinations that I have ever used. Of course this solution may be diluted, according to the indications of the particular case and the time of using it. The important part, however, in the use of this as well as any injection, is to properly inject it with a good syringe of small size, and to prevent the liquid from flowing out before its effect is produced. If the urine is voided previous to the injection which should be repeated at least four or five times a day or oftener, or if the urethra is first injected with simple water, to thoroughly cleanse it, a single syringe full is quite sufficient at one operation.

But is this all that is necessary in the treatment of gonorrhœa? In some cases it is, but in the majority of cases the internal administration of an alkali is very important and necessary in order to neutralize the acidity of the urine, and render it less irritating and scalding as it passes out of the urethra. Aside from this, however, there are other benefits derived from the continuous administration of an alkali in this as well as some other inflammatory diseases. It dissolves the fibrin of the blood and to some extent retards its formation, and in this way probably prevents a very troublesome chordee, which, as before stated, is caused by the effusion of coagulable matter into the meshes of the corpus spongiosum. In many cases, especially if the inflammation be very acute, it is a good practice to give an alkaline cathartic in the beginning of the disease, and repeat it if necessary. Finally, rest in bed if practicable, light wrapping over the penis, cold applications if there be epididymitis or chordee, and in case of the latter, camphor and opium, or hyoscyamus or lupulin may be given with good effect.

An unstimulating diet, the avoidance of all alcoholic stimulants, and everything that tends to induce sexual excitement, together with the continuation of the treatment for a few days or a week after the discharge has apparently ceased, will in the majority of cases accomplish the desired end.

For the treatment of the disease in the female, where it is usually confined to the vagina, stronger injections may be used if desirable.

